# Delivering online cognitive behavioral therapy to address mental health challenges in correctional workers: a randomized controlled trial

**DOI:** 10.3389/fpsyt.2026.1922912

**Published:** 2026-10-16

**Authors:** Christina Holmes, Gilmar Gutierrez, Callum Stephenson, Elnaz Moghimi, Niloufar Malakouti, Jasleen Jagayat, Charmy Patel, Alexander Simpson, Cory Gerritsen, Michael Martin, Mark Fitzpatrick, Jazmin Eadie, Anika Agrawal, Vineeth Jarabana, Kimia Asadpour, Ferwa Khan, Dianne Groll, Mohsen Omrani, Nazanin Alavi

**Affiliations:** 1 Department of Psychiatry, Faculty of Health Sciences, Queen’s University, Kingston, ON, Canada; 2 Department of Clinical Psychology, School of Health in Social Sciences, The University of Edinburgh, Edinburgh, United Kingdom; 3 Department of Psychiatry, University of Alberta, Edmonton, AB, Canada; 4 Waypoint Research Institute, Waypoint Centre for Mental Health Care, Penetanguishene, ON, Canada; 5 Department of Psychiatry, University of Toronto, Toronto, ON, Canada; 6 Centre for Neuroscience Studies, Faculty of Health Sciences, Queen’s University, Kingston, ON, Canada; 7 Department of Forensic Psychiatry, Center for Addiction and Mental Health, Toronto, ON, Canada; 8 Graduate Department of Psychological Clinical Sciences, University of Toronto, Toronto, ON, Canada; 9 Health Services Sector, Correctional Service of Canada, Ottawa, ON, Canada; 10 School of Epidemiology and Public Health, University of Ottawa, Ottawa, ON, Canada; 11 Department of Psychology, Carleton University, Ottawa, ON, Canada; 12 OPTT Inc., Toronto, ON, Canada; 13 Faculty of Medicine and Health Sciences, McGill University, Montreal, QC, Canada; 14 Centre for Neuroscience Studies, Kingston Health Sciences Centre, Kingston, ON, Canada; 15 Centre for Digital Health Research (CDHR), Providence Care Hospital, Kingston, ON, Canada

**Keywords:** correctional workers, digital mental health interventions, internet, mental health, mental health disorders, online, online cognitive behavioral therapy, participant experiences

## Abstract

**Background:**

Correctional workers (CWs), are frequently exposed to traumatic workplace events, placing them at higher risk of experiencing mental health disorders, including major depressive disorder (MDD), generalized anxiety disorder (GAD), and posttraumatic stress disorder (PTSD). Despite high prevalence of mental health challenges, CWs underutilize mental health services due to stigma, irregular work schedules, and accessibility issues. While cognitive behavioral therapy (CBT) is effective, it poses accessibility challenges. Electronic CBT (eCBT) offers a scalable, accessible alternative, but use among CWs remains underexplored.

**Objective:**

This study aimed to evaluate the efficacy of diagnosis-specific eCBT programs designed for CWs in reducing symptoms of MDD, GAD, and PTSD compared to treatment as usual (TAU).

**Methods:**

A randomized controlled trial (RCT) was conducted with 84 CWs in Ontario, assigned to either eCBT (n=40) or TAU (n=44). The eCBT program provided diagnosis-specific modules with synchronous personalized feedback from care providers over 12-weeks via the Online Psychotherapy Tool (OPTT). Symptom severity was measured at baseline, week-6, and post-treatment using validated scales for depression, anxiety, PTSD, and quality-of-life. Data analysis included paired samples t-tests, mixed linear models, and effect size calculations (Cohen’s d).

**Results:**

The eCBT group exhibited significant reductions in symptom severity for depression, anxiety, and PTSD from baseline to post-treatment compared to TAU (p<0.01), with a moderate effect size (Cohen’s d=0.68). Symptom severity decreased by 61.37% for eCBT versus 23.29% in TAU. Mixed linear models confirmed a significant treatment-by-time interaction, favoring eCBT (p<0.01). However, no significant differences in quality-of-life improvements were observed between groups.

**Conclusions:**

Diagnosis-specific eCBT programs are effective in reducing symptoms of depression, anxiety, and PTSD for CWs. The asynchronous, accessible format of eCBT addresses current key barriers. Future research should explore strategies to improve adherence and increase the accessibility of services for CWs.

## Introduction

Public safety personnel (PSP), including correctional workers (CW), often experience adverse mental health outcomes associated with repeated exposure to traumatic work events (1). These can include direct or indirect experiences violence, injury, or threats to life, which significantly increase their risk of developing various mental health problems compared to the general population (1–4). Despite significant vulnerability, mental health service utilization is low in this population (5, 6). Systematic factors such as negative stigma and demanding work schedules are significant barriers to treatment-seeking (5, 6). Given the high prevalence of mental health issues, as well as the challenges to treatment-seeking, developing innovative solutions to address mental health within the CW population should be a public safety priority (7).

PSPs experience a uniquely stressful work environment which includes regular experiences of threats to personal safety, inmate violence or deaths, and direct injury (8, 9). The Union of Canadian Correctional Officers (UCCO) found that 98% of CWs have faced at least one traumatic event at some point in their career, with about half experiencing an assault on a colleague or the death of an inmate (10–12). PSPs are 3-times more likely to develop a mental health disorder, including post-traumatic stress disorder (PTSD), major depressive disorder (MDD), and generalized anxiety disorder (GAD) and four times more likely to experience suicidal ideation, attempts, and deaths compared to the general population (2–4, 12). Those seeking support face unique barriers to care, including irregular shift hours, understaffing issues, fear of professional repercussions, and financial costs (5, 13).

The frequent traumatic work experiences and increased mental health needs of CWs require specialized therapeutic care. Cognitive behavioral therapy (CBT) is a widely practiced and accepted intervention for various mental health disorders, including MDD, GAD, and PTSD (6, 7, 14). CBT has shown effectiveness in managing various mental health symptoms and reducing workplace absenteeism, particularly when tailored for workplace situations (15, 16). CBT has also been shown to be an effective treatment for PTSD symptoms in PSPs with long-lasting effects (17, 18). Despite the well-established effects of CBT, barriers such as privacy concerns, high costs, and poor accessibility, availability, and scalability impact help-seeking (5–7, 19). Electronic CBT (eCBT) has emerged as a promising and effective solution to providing an accessible, scalable, and convenient treatment option that patients can access from their homes (5–7, 19, 20). Both CBT and eCBT utilize practical, evidence-based therapeutic approached that focus on how thoughts, feelings and behaviors connect and affect each other, however eCBT is delivered via digital platforms to enhance accessibility and scalability to groups with significant barriers to care such as CWs. Given the treatment barriers experienced by PSPs, these attributes make eCBT a fitting approach to address their mental health problems (5–7, 19, 20). Nevertheless, though the effectiveness of eCBT interventions has been well validated in the literature, eCBT directly targeted at CWs is scarce and understudied (5–7, 18, 19). The chronic workplace trauma that CWs face makes their experience unique, requiring a more specialized eCBT approach. Traditional eCBT does not integrate the experiences faced by CWs, leaving no eCBT specifically designed for CWs.

This research team has previously developed and validated various eCBT modules to address a myriad of mental health disorders, including MDD, GAD, and PTSD (6, 7). Considering vulnerability and systemic barriers, this study focused on creating diagnosis-specific eCBT programs tailored to CWs. The adaptations to standard eCBT were guided by the lived-experiences of CWs (7, 21), these adaptations included tailored language to better address occupational barriers and stressors such as CW specific story examples in the eCBT modules generated through focus groups with CWs (see study protocol for more information; 7). This paper presents the quantitative findings, examining the efficacy of the designed modules.

## Materials and methods

### Study design

A comprehensive explanation of the protocol for this study has been published previously (7). This study was a randomized controlled trial (RCT) comparing the effectiveness of an eCBT program (experimental group) to treatment as usual (TAU; control group) for the management of MDD, GAD, or PTSD symptoms in CWs. According to current research, TAU for this population may included standard medical check-ups, general support or peer support and baseline self-directed lifestyle without additional structure psychotherapy (22). Those in the eCBT arm received diagnosis-specific modules for 12–13 weeks with personalized feedback from a care provider. Those in TAU continued with current treatment methods with no additional intervention. For the purpose of this study, no additional information on TAU was collected, but participants were excluded if they had participated in another CBT program in the past six-months,. Upon providing verbal consent to participate in this study, participants were randomized into either the eCBT or the control arm. Randomized allocation was carried out using a computerized random number generator by a team member who was not associated with data collection or analysis. Before starting their participation in the study, participants received a demographic questionnaire to collect their age, biological sex, ethnicity, marital status, employment status, income, and education level (7). For the purpose of this study, employment status refers directly to most recent role if currently retired. This study received ethical clearance from the Queen’s University Health Sciences Research Ethics Board (File: 6029966). This trial was registered through Clinicaltrials.gov (NCT04666974).

### Sample size and recruitment

The sample size was determined based on previous results in the literature showing that eCBT had a significant effect in the reduction of depression (Hedges’ G = 0.78), anxiety (Hedges’ G = 0.83), and PTSD (Hedges’ G = 0.51) symptoms in PSP (18). Considering a power of 80% and a significance of p=0.05, a sample of 25 in each arm was determined to be suitable for detecting a significant effect in a paired sample t-test. Thus, accounting for an estimated completion rate of 45% from previous studies, we aimed to recruit 55 participants in each arm (n=110) (7, 19, 23).

Recruitment was conducted across Ontario through self-referral or via primary care providers, using flyers, social media, and union outreach. Eligible participants were current or former correctional workers, aged 18+ with symptoms of MDD, GAD, or PTSD per DSM-5 criteria (7, 24, 25). Inclusion criteria considered competency to provide informed consent, English fluency, and consistent internet access. Exclusion criteria included participation in another CBT program in the past six-months, active hypomanic/manic symptoms, psychosis, severe alcohol or substance use, and acute suicidal or homicidal ideation. Medication use was permitted if stable.

Recruited participants underwent a comprehensive initial mental health assessment by a psychiatrist on the research team. Diagnoses were identified using the structured Mini-International Neuropsychiatric Interview (MINI) performed by a trained research assistant and a psychiatric assessment conducted by a psychiatrist on the research team. The MINI diagnostic tool does not use cut-off scores as you may see with traditional questionnaires. Instead, this method uses algorithmic branching based off of clinical cut-offs for binary question responses. This method allowed us to differentiate overlapping symptoms between different diagnosis’. Participants with comorbid symptoms were assigned to the track matching their principal diagnosis or most disabling symptoms based on psychiatric assessment.

### Interventions

*Experimental Group – Diagnosis-Specific eCBT Program + Treatment as Usual (TAU):* The eCBT program mirrored validated traditional in-person CBT sessions to deliver pre-designed interactive therapeutic content tailored to the unique circumstances experienced by CWs and PSPs. Focus groups composed of a diverse group of CWs helped develop programs, which emphasized coping strategies and cognitive-behavioral techniques relevant to occupational challenges (7, 21). If the participants were randomized to the experimental group, they were assigned to one of three diagnosis-specific eCBT tracks—MDD, GAD, or PTSD—based on clinical symptoms.

The 12-week eCBT modules are designed to mirror traditional CBT therapeutic tasks. Each interactive session consists of approximately 30 slides and interactive videos covering standard CBT concepts (such as the connection between thoughts, feelings, behaviors, physical responses, and environment). Every session concludes with a mandatory clinical homework assignment in which the participants must complete by an agreed upon time with their care provider. Participants are required to submit this assignment through the online portal by a set deadline to unlock the next sequential module. A weekly assignment might include a Thought Record log in which participant will log a distressing automatic thought, identify the cognitive distortion (e.g., catastrophizing), and construct an alternative, balanced thought.

Care providers supported participants asynchronously by reviewing their assignments, and providing personalized feedback (7, 19, 23). The clinician spends approximately 15 minutes drafting individualized therapeutic feedback using a structured, pre-validated template that ensures consistent care delivery while remaining specific to the participant’s lifestyle and profession. This response delivered directly to the patient alongside their next module. ECBT was delivered through OPTT, a secure, cloud-based online care platform hosted through Amazon Web Services Canada, managed by Medstack. OPTT complies with the Health Insurance Portability and Accountability Act (HIPAA), Personal Information Protection and Electronic Documents Act (PIPEDA), and Service Organization Control-2 (SOC-2) and collects anonymized metadata to enhance platform performance (7, 19, 23). This group continued receiving TAU that is conducted outside of the research study.

*Control Group:* Participants randomized to the control group received the same psychiatric scales as those in the experimental group for 12 weeks. After 12 weeks, they had the option of receiving the eCBT program most suitable for their diagnosis and symptoms.

### eCBT care providers

Care providers in this study were mental health research assistants, trained to deliverpsychotherapy under the supervision of the PI (licensed psychotherapist, expert in eCBT). Researchassistants all had a minimum bachelor’s degree in a related subject and underwent ongoingtraining and supervision by the PI throughout the course of the study. Training to deliver therapyin this study consisted of a psychotherapy workshop and dedicated CBT learning sessions. Tostandardize the quality of the care, personalized feedback written by the care providers followedpre-designed feedback templates, which were reviewed by the PI before delivery. Using these feedbacktemplates (see **Appendix 1**), the care providers were able to write the personalized feedback in 15–20 minutes (7, 19, 23).

### Outcome measures

Demographic information such as age, biological sex, ethnicity, marital status, income, and education level, was collected prior to engagement using a questionnaire integrated in OPTT. The primary outcome measure involved assessing the effectiveness of the eCBT program compared to the control group by tracking symptom changes. Symptomatology changes in both arms were assessed at baseline (week 0), mid-intervention (week 6), and post-intervention (week 12) using validated scales for depression (Depression subscale of the Depression Anxiety Stress Scale; DASS-42, Quick Inventory of Depressive Symptomatology – Self-Report; QIDS-SR-16, and Patient Health Questionnaire; PHQ-9), anxiety (Anxiety subscale of the DASS-42, Generalized Anxiety Disorder 7-Item Questionnaire; GAD-7), stress (Stress subscale of the DASS-42), PTSD (PTSD Checklist 5-Item; PCL-5), and quality of life (Quality of Life Enjoyment and Satisfaction Questionnaire – Short Form; Q-LES-Q-SF) (26–31). Treatment response was defined as a total score reduction of 50% or more from baseline to study endpoint for the PHQ-9, DASS-42, QIDS-SR-16, GAD-7, and PCL-5 scales (32). For the Q-LES-Q-SF, treatment response was defined as a total score increase of 6.80% from baseline to study endpoint (33). The secondary outcomes measure involved determining the average number of sessions completed and dropout rates in the two treatment arms.

### Statistical analysis

All calculations for this study were conducted on IBM SPSS Statistics for Mac, version 25 (IBM Corporation, Armonk, NY, USA). All tests were two-tailed and evaluated at an uncorrected alpha of p<0.05, consistent with the pre-specified analysis plan in the published protocol (7). No adjustment for multiple comparisons was applied to the paired samples t-tests, independent samples t-tests, or mixed linear models, either across scales or across assessment time points. The reference to Bonferroni correction in the previous version of this section was carried over from the protocol in error and has been removed. Because no familywise correction was applied, scale-level and time-point-level p-values should be interpreted as exploratory, and the treatment-by-time interaction in the mixed linear model (**Table 1**) is the primary inferential test (34).

**Table 1:** Outcome analysis of linear unstructured mixed model displaying treatment x time interaction for symptom severity.

Symptom Severity	Treatment		Time		Interaction	
Symptom Severity	*F*	*p*	*F*	*p*	*F*	*p*
	1.78	0.19	25.85	<0.001	6.98	0.001

Before analysis, missing values were identified and removed, following a per-protocol approach. Descriptive statistics were analyzed to compare demographic variables and the baseline, midpoint and post-treatment symptomatology severity scores and quality-of-life scores between the experimental and control groups (26–31). Primary questionnaire scores were normalized by dividing each data point by the maximum possible score for that questionnaire. This allowed scores to be compared across diagnoses (PHQ-9 maximum score of 27; GAD-7 maximum score of 21; PCL-5 maximum score of 80). Paired samples t-tests were used to determine statistical significance. This study used Cohen’s D (d) to report the effect size of the descriptive statistical analysis with a 95% confidence interval (95% CI) (34, 35). The data was analyzed using linear regression to examine the relationship between psychiatric scale changes and assessment time points. ANOVA was used to explore the interaction between treatment effects and treatment group (eCBT or TAU), diagnosis-specific program (eCBT for MDD, GAD, or PTSD), assessment time points (0, 6, and 12 weeks), and demographic variables (i.e., age, biological sex, ethnicity, education level, etc.). For ANOVA, this analysis assumed data normality, homogeneity of covariance matrices, and linearity. Finally, several completed sessions and dropout data were expressed as a proportion to compare eCBT to TAU (36). An attrition analysis was conducted to comparing clinical characteristics across completers and dropouts, which were analyzed separately within each arm. The data generated or analyzed is available as needed upon reasonable request.

## Results

### Participants

Eligible participants (n=84) were randomized into treatment (n=40) or control (n=44) (**Figure 1**). Although our original aim was to recruit 55 participants in each arm, the sample was smaller-than-proposed sample due to time constraints and recruitment limitations. Participants were middle-aged (eCBT: M = 42.04, SD = 9.74; Control: M = 55.56, SD = 10.05). All participants were fluent in English, with majority native speakers (eCBT=97.73%; Control=96.30%). Majority of participants were born in Canada (eCBT=94.74%, Control=92.59%). Participants in the eCBT arm completed an average of 6.48 (SD = 4.58) sessions, including those who first were allocated to the control group then transferred to eCBT. Dropout rates across both groups were high (54.5% in control and 65% in eCBT); this is similar to dropout patterns seen in current psychiatric research. Given the attrition rates observed in both groups, comprehensive attrition analyses were conducted to evaluate potential selective attrition bias based on categorical traits. Due to low-frequency cell counts in all variables, Fisher’s exact tests were uniformly applied across all categorical variables to ensure statistical exactness. Analyses were executed independently for each treatment arm.

**Figure 1:**
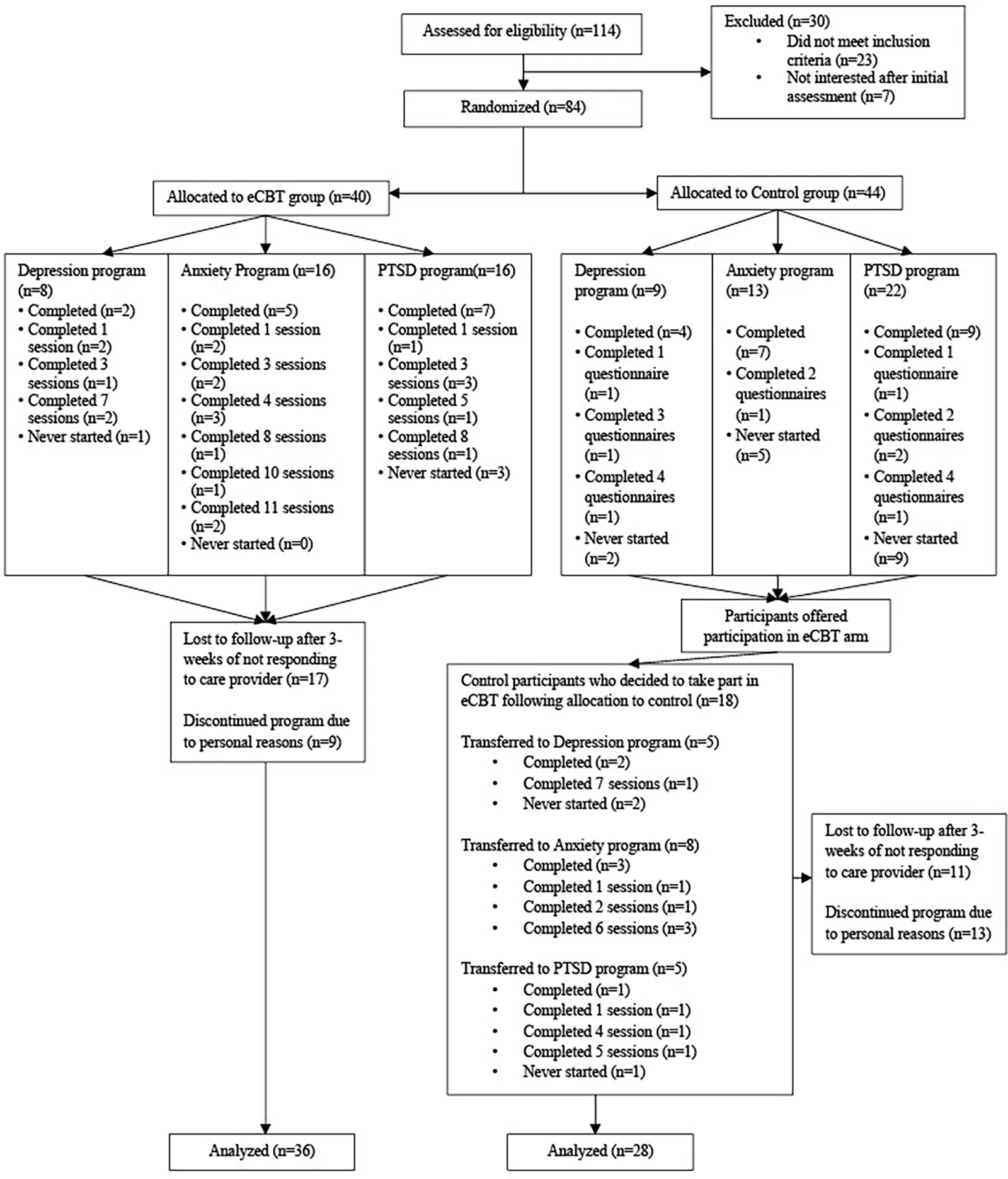
Consort diagram.

Within the eCBT arm, completion status (defined as attending al 12 sessions) was not significantly associated with any categorical demographic (all *p* >.05). Similarly, within the control arm, no statistically significant differences emerged between completers and dropouts across any evaluated category (all p >.05). These non-significant findings demonstrate that attrition occurred evenly across all subgroups, confirming that dropout was disproportionately driven by any specific baseline demographic or diagnosis. Full categorical distributions and exact p-values are detailed in **Table 2**.

**Table 2:** Participant sociodemographic characteristics and side effect profile by study arm.

Category & response count (n)	eCBT n (%)				Control n (%)			
	Total (n=59)	Completer (n=18)	Dropout (n=41)	Fisher’s *p*	Total (n=46)	Completer (*n* = 20)	Dropout (*n* = 26)	Fisher’s *p*
Gender Identity	n=20				n = 9			
*Female*	12 (60.00)	4 (33.33)	8 (66.66)	0.36	5 (55.56)	3 (60.00)	2 (40.00)	0.44
*Male*	8 (40.00)	5 (62.50)	3 (37.50)		4 (44.44)	4 (100.00)	0 (0.00)	
Sex	n=45				n=34			
*Female*	19 (42.22)	7(36.84)	12 (63.16)	1	9 (26.47)	6 (66.66)	3 (33.33)	1
*Male*	26 (57.78)	10 (38.46)	16 (61.54)		25 (73.53)	14 (58.33)	10 (41.67)	
Race/Ethnicity	n=44				n=27			
*White (Caucasian)*	32 (72.73)	11(34.38)	21 (65.62)	0.48	24 (88.89)	17 (70.83)	7 (29.17)	1
*Asian*	0 (0.00)	0 (0.00)	0 (0.00)		1 (3.70)	1 (100.00)	0 (0.00)	
*Metis*	1 (2.27)	0 (0.00)	1 (100.00)		2 (7.41)	1 (50.00)	1 (50.00)	
*Other*	11 (25.00)	6 (54.55)	5 (45.45)		0 (0.00)	0 (0.00)	0 (0.00)	
Marital Status	n=44				n=27			
*Married*	27 (61.36)	12 (44.44)	15 (55.56)	0.89	20 (74.07)	14 (70.00)	6 (30.00)	0.55
*Never married*	9 (20.45)	3 (33.33)	6 (66.66)		2 (7.41)	2 (100.00)	0 (0.00)	
*Divorced*	5 (11.36)	1 (20.00)	4 (80.00)		3 (11.11)	2 (66.66)	1 (33.33)	
*Widowed*	1 (2.27)	0 (0.00)	1 (100.00)		0 (0.00)	0 (0.00)	0 (0.00)	
*Other*	2 (4.55)	1 (50.00)	1 (50.00)		2 (7.41)	2 (100.00)	0 (0.00)	
Children	n=43				n=26			
*Yes*	26 (60.47)	12 (46.15)	14 (53.85)	0.33	16 (61.54)	11 (73.33)	4 (26.67)	1
*No*	17 (39.53)	5 (29.41)	12 (70.59)		10 (38.46)	8 (80.00)	2 (20.00)	
*1 Child*	9 (40.91)	3 (33.33)	6 (66.66)	0.22	3 (20.00)	3 (100.00)	0 (0.00)	0.71
*2 Children*	7 (31.82)	5 (71.42)	2 (28.58)		4 (26.67)	2 (50.00)	2 (50.00)	
*3 Children*	4 (18.18)	2 (50.00)	2 (50.00)		7 (46.67)	4 (57.14)	3 (42.86)	
*4 Children*	1 (4.55)	1 (100.00)	0 (0.00)		0 (0.00)	0 (0.00)	0 (0.00)	
*5 Children*	1 (4.55)	1 (100.00)	0 (0.00)		1 (6.67)	1(100.00)	0 (0.00)	
Education	n=44				n=25			
*High School Diploma*	1 (2.27)	0 (0.00)	1 (100.00)	0.17	0 (0.00)	0 (0.00)	0 (0.00)	0.56
*College Diploma*	15 (34.09)	3 (20.00)	12 (80.00)		15 (60.00)	5 (33.33)	10 (66.66)	
*Bachelor’s Degree*	24 (54.55)	11 (45.83)	13 (54.17)		10 (40.00)	5 (50.00)	5 (50.00)	
*Graduate Degree*	4 (9.09)	3 (75.00)	1 (25.00)		0 (0.00)	0 (0.00)	0 (0.00)	
Employment Status	n=44				n=27			
*Full-Time*	41 (93.18)	17 (41.46)	24 (58.54)	0.70	25 (92.59)	18 (72.00)	7 (28.00)	0.46
*Part-Time*	1 (2.27)	0 (0.00)	1 (100.00)		1 (3.70)	0 (0.00)	1 (100.00)	
*Unemployed*	2 (4.55)	0 (0.00)	2 (100.00)		1 (3.70)	1 (100.00)	0 (0.00)	
Annual Income	n=44				n=25			
*Over $100,000*	13 (29.55)	6 (46.15)	7 (53.85)	0.60	8 (32.00)	3 (37.50)	5 (62.50)	1
*$75,000 to $99,999*	25 (56.82)	8 (32.00)	17 (68.00)		12 (48.00)	5 (41.67)	7 (58.33)	
*$50,000 to $74,999*	6 (13.64)	3 (50.00)	3 (50.00)		4 (16.00)	2 (50.00)	2 (50.00)	
*Under $50,000*	0 (0.00)	0 (0.00)	0 (0.00)		0 (0.00)	0 (0.00)	0 (0.00)	
*Disability Pay*	0 (0.00)	0 (0.00)	0 (0.00)		4 (4.00)	0 (0.00)	4 (100.00)	
Diagnosis	n=59				n=46			
*Depression*	14 (23.73)	4 (28.57)	10 (71.43)	1	11 (23.91)	4 (36.36)	7 (63.64)	0.76
*Anxiety*	24 (40.68)	7 (29.17)	17 (70.83)		13 (28.26)	7 (53.85)	6 (46.15)	
*Posttraumatic Stress Disorder*	21 (35.59)	7 (33.33)	14 (66.67)		22 (47.83)	9 (42.86)	12 (57.14)	

*Education categories are in reference to Canadian educational standards and may differentiate to international qualifications.

A significantly higher proportion of control participants were White (p=0.02). However, all other demographic variables were comparable between the two arms. There was a large percentage of unanswered questions in the category of ‘gender identity’ in the sociodemographic questionnaire. This must be considered when interpreting the data.

### Clinical outcomes

Percent change and normalized score change the main questionnaires in each diagnosis was analyzed to determine the effectiveness of each arm on their respective symptom severity outcomes (PHQ-9 for depression, GAD-7 for anxiety and PCL-5 for PTSD). Paired sample t-tests revealed significant improvements in the e-CBT arm post-treatment in PCL-5 and DASS-42 scores. At the midpoint, PHQ-9, GAD-7, and DASS-42 scores showed statistical improvements. There were no statistically significant improvements in the control group.

At baseline, independent sample t-tests revealed a significantly higher DASS-42 (p<0.03) score in the treatment group. However, no difference in baseline was observed for all other scores, including normalized symptom severity (t=1.34, p=0.19). At the midpoint, questionnaire scores did not differ between the treatment and control arms (t=-1.50, p=0.14). The participants’ questionnaire scores for their main diagnosis’ at each time point can be found below in **Table 3**. Although all participants completed each questionnaire at baseline, mid-point and completion; this table highlights the respective symptom severity outcomes for each individual group as well as quality of life responses across all groups. **Table 3** selectively reports the primary diagnostic scale corresponding to each specific disorder to isolate condition-specific symptom changes, rather than reporting all administered secondary measures.

**Table 3:** Mean questionnaire scores and standard deviations for different diagnoses in both arms, as well as effect size (Cohen’s d) and significance.

Measure	Time point	eCBT (mean, SD)	Control (mean, SD)	Cohen’s d (95% CI)	p-value
*PHQ-9*	T0	10.88 (5.21)	10.42 (5.54)	0.09 (-0.34, 0.51)	0.70
	T1	8.68 (4.49)	8.68 (4.49)	0.00 (-0.43, 0.43)	1.00
	T2	8.24 (5.82)	9.70 (5.52)	-0.26 (-0.69, 0.17)	0.25
*GAD-7*	T0	10.61 (5.48)	9.35 (4.34)	0.26 (-0.17, 0.69)	0.25
	T1	7.94 (5.08)	8.91 (4.48)	-0.20 (-0.63, 0.23)	0.36
	T2	8.10 (5.10)	9.30 (5.06)	-0.23 (-0.65, 0.19)	0.28
*PCL-5*	T0	32.96 (17.23)	31.28 (14.34)	0.10 (-0.31, 0.52)	0.64
	T1	27.24 (17.03)	30.47 (14.95)	-0.19 (-0.62, 0.24)	0.38
	T2	19.45 (16.37)	27.95 (18.20)	-0.49 (-0.91, -0.07)	0.02*
*Q-LES-Q-SF*	T0	42.54 (8.58)	43.00 (7.67)	-0.06 (-0.48, 0.36)	0.79
	T1	44.76 (8.88)	42.18 (8.15)	0.30 (-0.12, 0.72)	0.16
	T2	46.95 (10.56)	42.15 (8.77)	0.51 (0.09, 0.93)	0.02*

A decrease in the symptomatology questionnaire score represents an improvement in symptom severity. An increase in the quality of life score represents an improvement in quality of life. Significant outcomes are marked with an asterisk (*). T0 = Baseline, T1 = Midpoint, T2 = Post-Treatment.

Paired samples t-tests revealed a two-tailed statistically significant improvement from baseline to midpoint in the eCBT group for PHQ-9 (p<0.01), GAD-7 (p<0.01), and DASS-42 (p<0.01) scores. From baseline to post-treatment, a statistically significant improvement in PCL-5 (p<0.05) and DASS-42 (p<0.01) scores was found in the eCBT group. Additionally, at post-treatment, the eCBT arm had a significantly lower normalized questionnaire score (t=-2.78, p<0.01) and a moderate effect size (Cohen’s d=0.68). There were no significant differences between the two arms regarding quality-of-life scores or percent change of quality of life at any time point. Percent changes from baseline to midpoint (week 6) and baseline to post-treatment (week 12) can be found in **Table 4**. Mixed linear models showed, with an intention-to-treat (ITT) analysis, that there was a significant treatment-by-time interaction (p<0.01) regarding symptom severity improvement in the eCBT arm (**Table 1**; **Figure 2**).

**Table 4:** Percent changes across time points for primary questionnaire scores.

Group	T0 - T1	T0 - T2
eCBT		
*Symptom Severity*	-36.87%	-61.37%
*Quality of Life*	4.50%	6.96%
Control		
*Symptom Severity*	-19.03%	-23.29%
*Quality of Life*	0.06%	0.82%

A negative change in the merged symptom severity score indicates an improvement. An increase in the quality of life score indicates an improvement. T0 = Baseline, T1 = Midpoint, T2 = Post-Treatment.

**Figure 2:**
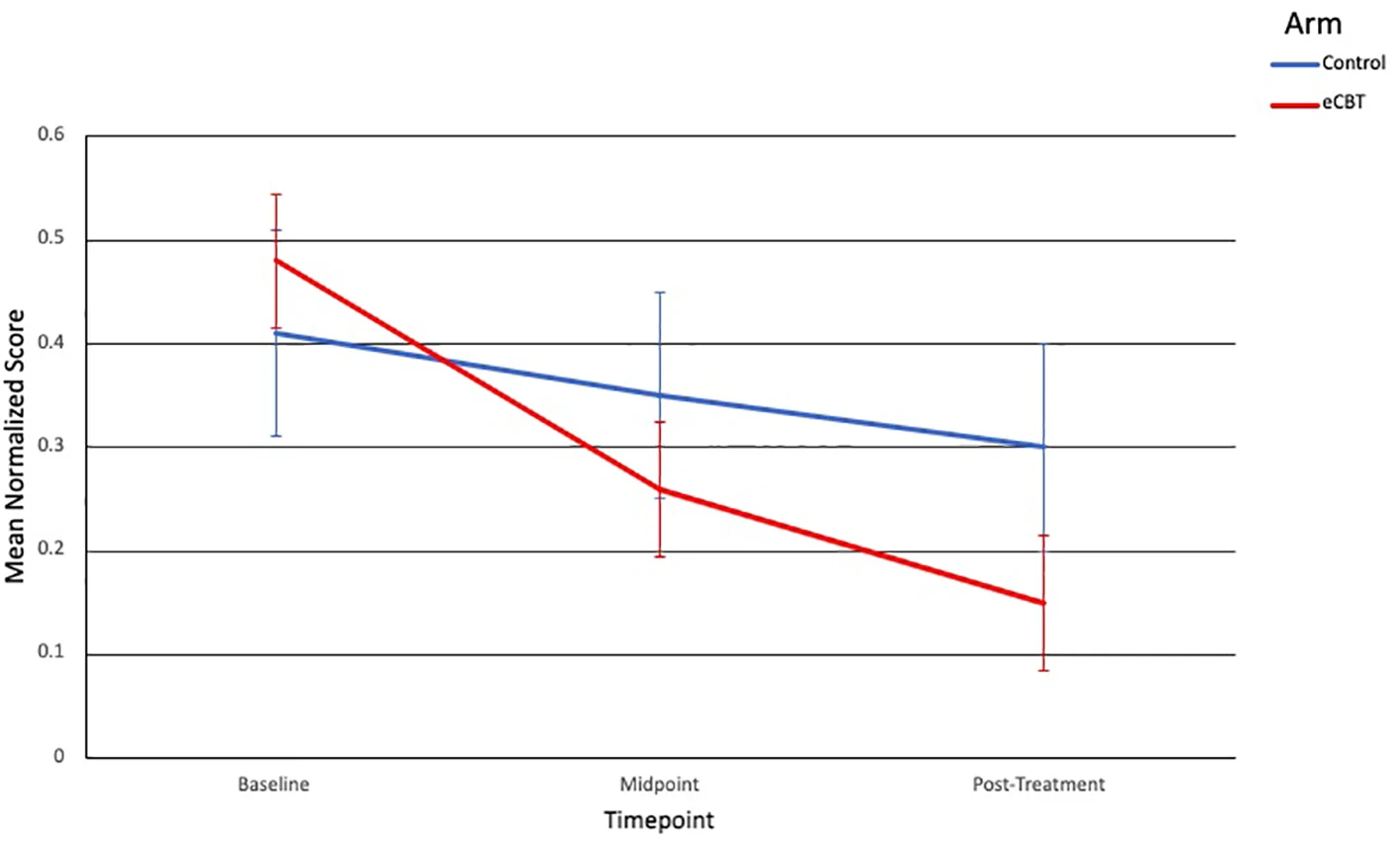
Mixed linear model displaying treatment x time interaction with a modified ITT analysis.

## Discussion

The findings of this study suggest that eCBT is a promising intervention for reducing symptom severity among CWs. CWs who received eCBT reported significantly lower questionnaire scores and a moderate-effect size post-treatment. Symptom severity from baseline to post-treatment demonstrated a large improvement. However, many participants only completed approximately half of the sessions. More participants completed the questionnaires at the midpoint than post-treatment, which must be considered when interpreting results. Overall, these findings suggest that eCBT is effective in treating depression, anxiety, and PTSD, especially when the program is completed. A modified ITT analysis, including participants who completed at least one post-baseline assessment (eCBT: n=36; Control: n=30), revealed a significant treatment-by-time interaction in the eCBT arm (p < 0.01), indicating notable symptom reduction. No such interaction was observed in the control arm. This model further showed that the eCBT arm reported lower symptom severity at the midpoint and post-treatment when compared to the control.

A modified ITT approach was applied due to missing post-baseline data, which preserved ITT advantages. These results align with previous evidence supporting eCBT for various mental health concerns (5–7, 19, 20) and underscore its value for CWs coping with occupational stressors (3, 7). Given prevalent barriers like shift work and stigma, eCBT may improve mental health access—though further research is needed to enhance adherence and refine delivery.

Our findings are consistent with the current literature surrounding the efficacy of eCBT for PSP populations. Significant improvements in anxiety and depression symptoms following an eCBT program within a population of police officers were reported (37). These findings suggest that eCBT may be effective in treating various mental health symptoms in high-risk, high-stress populations who have increased exposure to workplace trauma or violence, similar to that of CWs. Furthermore, eCBT was found to significantly reduce PTSD symptoms among military personnel (38), further highlighting the potential benefits of this type of treatment for populations with repeated exposure to stressful and traumatic work events.

A notable pattern present in this study is the differential timing of treatment effects across diagnostic domains with depression and anxiety symptoms improving earlier in the eCBT arm, while PTSD symptoms showed significant change only by post-treatment. This difference is consistent with established CBT mechanisms and the clinical course of trauma-related symptom change. Early improvements in depression and anxiety likely reflect the front-loaded structure of CBT, which introduces foundational skills, such as cognitive restructuring, behavioral activation, and anxiety-management strategies, within the initial modules (14–16). In contrast, PTSD symptoms typically require more time-intensive therapeutic processes, including trauma-focused cognitive restructuring, exposure-based work, and gradual re-engagement with avoided memories or situations. These components generally appear later in CBT treatments and require sustained engagement to produce measurable change. This aligns with the finding that PCL-5 scores showed significant improvement only from baseline to post-treatment, whereas PHQ-9 and GAD-7 scores demonstrated significant change by week 6. Similar delayed PTSD responses have been reported in PSP and military populations undergoing CBT or eCBT reflecting the complexity of trauma-related symptoms (17, 18, 37, 38).

Additionally, the lack of significant between-group differences in quality-of-life (QoL) outcomes warrants consideration. QoL measures capture broad domains such as occupational functioning, interpersonal relationships, and environmental stressors. often extending beyond symptom severity. For correctional workers, these domains are heavily influenced by persistent workplace stressors such as irregular shifts, understaffing, and exposure to traumatic events, all of which may limit the immediate improvements in QoL. Prior research similarly shows that QoL often is not improved parallelly to symptom improvement, particularly in populations experiencing chronic occupational stress (3, 7, 12). These findings suggest that substantial reductions in depression, anxiety, or PTSD may not produce immediate changes in broader life satisfaction if external stressors remain unchanged. This study further supports the notion that diagnosis-specific eCBT produces clinically meaningful symptom reductions, especially for depression and anxiety, PTSD symptoms and QoL may require longer treatment duration or adjunctive interventions to achieve comparable gains. Future research incorporating extended follow-up periods or hybrid treatment models may help clarify the trajectory of QoL change in correctional workers engaging in digital psychotherapy.

The tailored nature of the eCBT modules in this study may have contributed to the observed improvements in symptom severity. The modules developed for this study were generated specifically for the CW population and integrated the lived experiences of CWs into the content of the eCBT program. By utilizing focus groups with CWs, we were able to identify personal, social, and cultural factors uniquely reflective of the work of this population and developed diagnosis-specific programs (7). These specifications are necessary to capture the unique experiences faced by CW’s of populations as highlighted by the focus groups. This personalized approach may have facilitated a stronger therapeutic alliance and made the program more relevant to CWs, ensuring that the interventions were directly applicable to the real-world challenges faced by participants (7).

Alongside the personalized nature of the eCBT program, the asynchronous delivery of therapy within this study may allow for higher accessibility rates when compared to in-person CBT for this population. As many CWs face irregular shifts, understaffing, and tight work schedules, the online, asynchronous nature of the eCBT program allows for treatment to be accessed at convenient times for participants, overcoming time constraints and lack of immediate access to in-person therapists (6). Furthermore, the anonymity of the program may aid in reducing stigma associated with seeking mental health support. The stigma surrounding mental health is a well-established barrier to accessing treatment within high-stress occupations like CWs and often discourages individuals from seeking help due to fears of professional repercussions or being perceived as weak (12, 22). Online therapy, when tailored to the unique needs of a population, has been shown to result in high levels of satisfaction and engagement within more stigmatized professions (39). Future research should explore intervention methods to enhance compliance rates across high-risk populations.

### Limitations

The lack of compliance observed in both groups may be due to various factors, including stigma, discomfort in discussing mental health, and busy work schedules. However, treatment dropout is a common issue within the psychiatric populations in research and is like the findings of past research (40). Compliance rates remain a persistent challenge, especially in populations with demanding work environments, highlighting that this may be a larger concern for high-risk professions (8). While detailed compliance rate data for CW’s is limited, estimates from similar high-stress occupations remain consistent with the findings of this study and report completion rates of between 40-60% for in-person therapy and 60% of structured CBT programs, particularly when electronically delivered (8, 40, 41). Future work should investigate ways to address this low completion rate to allow CWs to benefit from eCBT.

Due to the highly sensitive nature of this occupation, recruitment efforts had to be made outside of correctional organizations. The inability to contact CWs directly through their organizations made recruitment difficult. To combat this issue, the research team contacted union groups, family physicians, and news sources to assist with recruitment efforts. Furthermore, following the program, many CWs reported being glad that their organizations were not involved in the advertisement process, and the separation from the workplace influenced their decision to participate (42). This further suggests that external pathways may be more effective in combatting barriers to treatment within this population. External referral pathways, Employee Assistance Programs (EAPs) and accessible services removed from organizational or institutional connections may be more likely to encourage engagement in this specific population. Due to these recruitment issues and the time constraints of the study, there was a smaller-than-proposed sample size, which may have reduced the statistical power of some analyses. The study originally aimed to recruit 110 participants, but only 84 were included in the final analysis, which limits the generalizability of the findings. Further research should aim to include larger sample sizes to ensure that the findings apply to the broader CW population.

Stigma remains a limitation to recruitment efforts, and it may affect how comfortable participants feel sharing vulnerable information and experiences with their care providers (43). The anonymity of the platform aimed to decrease stigma by making CWs feel more comfortable that their treatment is confidential. Integrating eCBT within EAPs or similar occupational health services could reduce these barriers by normalizing mental health support, providing confidential access, and embedding care within existing workplace structures. EAPs have been shown to improve help seeking among PSPs by offering rapid access, reduced financial burden, and protected confidentiality (7, 21). Further research should aim to look directly at the impact eCBT has on stigma for this population and future research should take this into account when constructing further care methods.

Moreover, the current study analyzed the combined results of the MDD, GAD, and PTSD programs. This may introduce confounding variables and mask possible nuances in how the respective eCBT programs impacted different diagnoses. A notable limitation is the assumption underlying our normalized scoring approach, which presumes that equivalent percentages of the maximum score across differing diagnostic instruments (PHQ-9, GAD-7, and PCL-5) reflect comparable levels of clinical severity. This specific normalization method was not independently validated within the current study. This is closely linked to the challenges of combining three distinct psychiatric diagnoses into a unified analysis, as identical percentage elevations may map onto differing levels of real-world functional impairment or diagnostic severity across the diagnostic tracks which cannot be distinguished. Additionally, symptom outcomes were assessed using multiple scales at three time points without adjustment for multiple comparisons. The nominal p-values for individual scale comparisons therefore carry an inflated familywise Type I error rate and should be interpreted as exploratory rather than confirmatory.

Furthermore, each eCBT program differed in content of the program and number of participants. 8 participants completed the PTSD and anxiety programs respectively, and 4 completed the depression program. This may result in the PTSD and anxiety groups being over-represented and may have impacted how these results are interpreted. These factors can add confounding variables and biases to our findings and must be considered when interpreting the results of the study. Future research should aim to analyze the eCBT programs independently to better understand how each tailored program performs for each specific disorder.

Ethnic diversity within the sample was low, potentially excluding unique experiences faced by CWs of minority backgrounds (44). While this may reflect trends of low racial diversity exists within the CW population (21), it limits generalizability and applicability to more diverse groups. Additionally, self-report measures are a potential source of bias, particularly in highly vulnerable populations like CWs (45). Participants may underreport symptoms due to workplace normalization of trauma, stress, or fear of judgement. Conversely, dissatisfaction with the work environment contributes to higher reports of psychological distress. These biases must be considered when interpreting results. Future research should continue utilizing personal experiences, including the unique circumstances of minority groups, to develop more efficient care. Medication usage was also a potential confounding variable. There was a high percentage of medication use in both the control and eCBT arms throughout the study, making it difficult to isolate the effects of eCBT. Although participants were asked to keep their medication stable, co-occurrence of therapeutic interventions complicates attribution of symptom changes. Future research should consider controlling for medication use or conducting analyses comparing medication-only and eCBT-only groups.

## Conclusion

CWs and PSPs continue experiencing mental health challenges due to frequent exposure to violence and harm in the workplace (1). These potentially traumatic events greatly increase the risk of developing mental health disorders (2–4). Systemic issues—including stigma and barriers to care—further complicate access to treatment and often discourage CWs from seeking help. Even when treatment is available, low adherence rates and internalized stigma reduce its effectiveness (2–4). Despite these challenges, the present study found eCBT to be a promising and effective therapeutic option. Diagnosis-specific eCBT programs, tailored to address CWs’ unique occupational stressors, showed improvements in symptom severity when compared to TAU and offered a more accessible intervention on a wider scale. These findings suggest that eCBT may be a viable alternative to address mental health concerns within CWs. The diagnosis-specific modules for depression, anxiety, and PTSD were effective in reducing symptoms across time points. Notably, participants who completed the eCBT sessions showed greater symptom reduction through treatment-by-time when compared to the control. Future work should develop more accessible and effective interventions for this specific population, building on the findings of this study to develop treatments that consider the unique experiences of CWs. Moreover, future work should aim to assess if greater tolerability for eCBT may increase compliance rates and decrease the negative stigma surrounding mental health treatments. The findings of this study should be considered when addressing current clinical practice for CWs and should be accounted for when responding to the current mental health needs of this population.

## Data Availability

The original contributions presented in the study are included in the article/**Supplementary Material**. Further inquiries can be directed to the corresponding author.
