# Supplementary material for: Delivering online cognitive behavioral therapy to address mental health challenges in correctional workers: a randomized controlled trial
Source: Front Psychiatry. 2026 Oct 16;17:1922912. doi: 10.3389/fpsyt.2026.1922912 (PMC13720534; doi:10.3389/fpsyt.2026.1922912)
Supplement: Supplementary file 1 [file DataSheet1.pdf]

Appendix 1: Feedback template example

Session 1: What is Depression?

Assignment Questions:

1. Tell me your story. What are your concerns at this time? What are your challenges? What are you hoping for?
1. Choose a SMART goal.
2. What is your small step?

Hello _________, *(participant's name)*

Thank you for sending me your assignment and sharing your story. I hope you find this course helpful and that the online format will suit you well. It might be difficult to share your emotions and what you are going through, and this itself can cause a lot of stress. Sharing your difficulties is a positive step that you have taken – I hope you can see this as an accomplishment.

Depression can make you feel very sad, and you may not enjoy your hobbies as much as before, your energy might be low, and you might notice changes in your sleep and appetite. These feelings might not go away for a long time. You might feel like you are all alone, worry about death, cry, or feel angry. Sadness can make your body feel different. This could include headaches, stomach aches, feeling sweaty or even nauseous or throwing up or having a pounding heart. Your body might feel really heavy and slow, and you might feel like you cannot make yourself do anything. If you take a step back and look at all the stressors in your life, you can see how they can cause you to feel depressed and stressed out. These stressors can cause you to experience different thoughts and some of them might be negative and unrealistic. We experience distorted thoughts when we have anxiety and/or depression. These thoughts will make you more depressed and as a result experience what you explained. Sometimes it might not be easy to connect the dots but if you look closely all these symptoms may be connected.

You discussed some challenges that you are currently facing and things you would like to improve on, such as *(include parts of 'Share your story.', summarize their challenges and depressive symptoms here)* how you feel about yourself and you would like to find a way to deal with stressful situations related to when your kids do something hurtful. That sounds very difficult to deal with _________ and I'm sorry you are going through this. You want to motivate yourself to physically go outside more and do activities, reconnect with old friends, and continue communicating with family and current friends, which you acknowledged that you have been neglecting through missed phone calls. I am glad that you were able to list the things you want to work on and slowly we can set them as achievable goals. Overall, you want to *(briefly summarize their goal(s) for themselves that they have listed, can use 'Specific' part of SMART goal here).* generally feel more joy and bring back the person you used to be by doing things, exploring, connecting with old friends, making new friends, and becoming a part of your friends and family's inner circle. I can tell that you are going through a lot, and it must not be easy. It seems like you are doing your very best and I can see that. I applaud you for trying and starting this program. We will work on these aspects throughout the course and help you get back to managing your day and feeling more like yourself!

There are tools in this course that we will go through that will help you break down and understand your thoughts, feelings, physical reactions, and behaviours, as well as learn how to change these negative thoughts into more effective ways of thinking. Together we can go through the stressful situations and overwhelming parts of your life and work through them so that you can handle stress better and feel more motivated. It's also good to think of a friend or family member that you find you can talk to about how you're feeling. Learning to communicate your feelings and just having someone that you feel comfortable

sharing your feelings and thoughts with can help ease some stress. We will work on these aspects throughout this course and help you get back to managing your day and feeling better _________.

In this session we discussed setting goals for therapy as an important factor in changing behaviour. Specifically, when determining a goal, we follow the SMART goal criteria. **SMART** is an acronym that helps us set up goals that are achievable and realistic. SMART goals are **Specific**: the goal is well defined and clear to anyone; **Measurable**: the goal allows for accurate tracking of progress so you know when you have met your goal; **Achievable**: the goal should be manageable for you; **Relevant and Realistic**: the goal should be applicable to the issue you would like to address and benefit you directly; and **Time-Based**: the goal should have a set timeframe to further quantify your goal and keep you on track.

Your **SMART** goal was centered around *(include 'Specific' part of SMART goal)* not laying around as much. I can see how it can be difficult for you to not lay around when you feel like you have low energy and when you are experiencing depression. To develop a SMART goal, it is helpful to be specific about what you are trying to work on. With a generic goal it can be hard to identify how you will know that you are completing it. *(provide feedback on the goal and 'specific' aspect as needed here (ex., if not specific enough, indicate and provide suggestions, etc).* You've chosen a great goal to set for yourself and this is something many people struggle with, but we will work on this together. This goal is measurable since you will *(include 'Measurable' part of SMART goal)* be able to not lay around as much and experience a change in your mood and energy levels as you start feeling better. You indicated this goal is achievable *(adapt this 'Achievable' part of SMART goal if needed)* and you have already started working on this goal by taking this course and seeking help. Good for you _________! It is realistic and relevant since it is something that will benefit you directly and you will *(adapt this 'Realistic/Relevant' part of SMART goal if needed)* have more energy once you start reaching your goal, allowing you to get things done around the house. Lastly, setting a timeframe for your goal is a good practice so that you know if you are on track. You mentioned a timeframe of *(adapt this 'Time-based' part of SMART goal as needed)* a couple of weeks. I'm hoping the 13 sessions of this course will help you achieve this goal, and we will work on this together! Your small step for this week was to *(include the small step part here)* not lay around as much. I'm sure this is easier said than done right now for you so maybe let's start off small and be a little bit more specific when deciding on a small step. What will you do instead of laying around (ex. I will go outside for some time today, I will make myself breakfast this week, I will start reading a book, listen to some music, etc.)? Find a small activity for you to do this week that can prevent you from laying around as much; it does not have to be big – something small works well, and we can start off with one thing this week. Overall, well done on providing a SMART goal and coming up with a small step!

I'm hoping the 13 sessions of this course will help you with your journey of achieving your goal, and we will work on this together! CBT is a very structured kind of therapy; it is the gold standard and the most effective evidence-based therapy for treatment of anxiety and depression. You will gradually see a connection between your thoughts, symptoms, and behaviours, and learn to change the way you think and behave in stressful situations, which will gradually improve your mood. CBT works specifically on challenging your thoughts and beliefs to a more effective way of thinking. As long as you are open and willing to learn the skills this course has to offer, we can slowly address your concerns and find tools that will help you in stressful situations. The purpose of the weekly questions at the end of the sessions is to help you apply and practice the techniques we learn in the session. If at any point you find that this program is not working for you and you would like to stop, or if you have thoughts on how we can support your progress, please don't hesitate to let me know. Like any therapy, collaboration can make a big difference. It can take some work to challenge some of your negative thoughts, but I am here to help you through this process, and I am hopeful that we will be able to work towards shifting your negative self-talk to something more effective.

I want to remind you that this program is designed to help you learn new techniques and strategies in stressful situations and is not designed for a crisis. If you experience suicidal ideation or feel that you need more help or support please call 911, or the 24-hour crisis line 613-544-4229, as we are not available all the time and we check the platform and messages once a week. We want to make sure that you are safe and get the best help you need and deserve.

** (If they asked any question in the questions section, don't forget to answer them at the end or integrate the answer in the feedback if that makes more sense).*

Together I'm hoping we can help you feel better and find the tools that work best for you! 😊 As long as you are open and willing to learn the skills this course has to offer, we can slowly address your concerns and find tools that help in stressful situations. I'm looking forward to working with you, ___________. Take care in the meantime.

Regards,

______________ (your name)
